# Enhancing adipogenesis in Wharton’s jelly multipotent mesenchymal stromal cells through lipidomic insights and fatty acid supplementation

**DOI:** 10.1038/s41598-025-16867-9

**Published:** 2025-08-22

**Authors:** Olena Rogulska, Eliska Vavrinova, Irena Vackova, Katarina Smolkova, Yuriy Petrenko

**Affiliations:** 1 Department of Neuroregeneration, Institute of Experimental Medicine, Czech Academy of Sciences, Prague, Czech Republic; 2 Laboratory of Biomaterials and Tissue Engineering, Institute of Physiology of the Czech Academy of Sciences, Prague, Czech Republic; 3 Department of Cryobiochemistry, Institute of Cryobiology and Cryomedicine, National Academy of Science of Ukraine, Kharkiv, Ukraine; 4 Charles University, Prague, Czech Republic; 5 Laboratory of Mitochondrial Physiology, Institute of Physiology of the Czech Academy of Sciences, Prague, Czech Republic

**Keywords:** Multipotent mesenchymal stromal cells, Adipogenic differentiation, Lipidomic profile, Wharton’s jelly, Adipose tissue, Triglycerides, Oleic acid, Linoleic acid, Cell biology, Adult stem cells, Mesenchymal stem cells, Stem-cell differentiation

## Abstract

Wharton’s Jelly multipotent mesenchymal stromal cells (WJ-MSCs) hold potential for regenerative medicine, particularly in soft tissue engineering. However, their adipogenic differentiation capacity is inferior to adipose tissue-derived MSCs (AT-MSCs). This study aimed to optimize adipogenic differentiation for WJ-MSCs by leveraging insights from the comparative analysis of WJ- and AT-MSC lipidomic profiles. Lipidomic profiles of non-induced cells were compared, and adipogenic differentiation was induced with and without exogenous oleic or linoleic acid supplementation. Differentiation efficiency was determined based on lipid droplet formation, triglyceride (TG) content quantification, and the expression of adipogenic markers. Significant differences in TG composition were observed, with WJ-MSCs showing higher levels of 52-carbon TGs and AT-MSCs having more 56-carbon species. Both cell types had similar fatty acid (FA) profiles, with 18-carbon FAs making up over 50%. Adding oleic acid to the differentiation medium significantly enhanced lipid droplet formation and upregulated adipogenic markers in WJ-MSCs, aligning their adipogenic capacity more closely with AT-MSCs. In contrast, linoleic acid showed no significant benefits. The study underscores the critical role of the initial lipidomic profile in the adipogenic differentiation of MSCs. Supplementation with oleic acid represents a promising approach for improving adipogenic differentiation of WJ-MSCs and their utility in soft tissue engineering.

## Introduction

Soft-tissue reconstruction remains a critical challenge in clinical practice, particularly in cases involving significant trauma, tumour resection, or congenital defects. These conditions often result in substantial functional and aesthetic impairments, necessitating advanced strategies for tissue restoration. Traditional approaches, such as autologous fat grafting and prosthetic implants, frequently yield suboptimal outcomes due to limitations such as graft resorption, donor-site morbidity, and inadequate biocompatibility. Addressing these shortcomings is essential for improving patient recovery and quality of life^1^.

Multipotent mesenchymal stromal cells (MSCs) have emerged as a promising tool in regenerative medicine due to their capacity for self-renewal, immunomodulation, and multilineage differentiation, including adipogenesis^2^. These unique properties make MSCs particularly suitable for applications in soft tissue reconstruction, where the generation of adipose tissue is critical for restoring volume and functionality^3,4^.

Among the various sources of MSCs, Wharton’s jelly-derived MSCs (WJ-MSCs) stand out for their distinctive advantages. These cells, isolated from the gelatinous connective tissue of the umbilical cord, exhibit high proliferative potential, low immunogenicity, and robust scalability for therapeutic applications^5,6^. Unlike MSCs from adipose or bone marrow tissues, WJ-MSCs’ functional potential is not influenced by donor age, and their isolation procedure is non-invasive and not linked to site morbidity, positioning them as a favourable option for allogeneic applications^5^. These features make WJ-MSCs particularly valuable in clinical scenarios requiring rapid and standardized cell-based therapies.

While MSCs derived from different tissue sources share a common immunophenotype, their functional properties – including differentiation potential, immunomodulatory capacity, and paracrine activity, may vary significantly depending on their origin. These differences are particularly evident in adipogenic differentiation^3^. Comparative studies have shown that adipose tissue-derived MSCs (AT-MSCs) exhibit an inherent predisposition for adipogenesis, likely due to their native lineage commitment and microenvironment^3,7,8^. However, AT-MSCs present important clinical limitations: their availability is often limited in patients with soft-tissue atrophy, volumetric loss, and tissue defects arising from lipodystrophy or reconstructive surgeries. In this case, WJ-MSCs offer a favourable alternative that overcomes the drawback of autologous cell use – scarcity of required tissue and patient-specific variability. Given these clinical advantages, WJ-MSCs represent a promising cell source for soft tissue engineering. However, their inferior adipogenic potential compared to AT-MSCs remains a significant barrier.

The difference in adipogenic differentiation between cell types is attributed to variations in gene expression profiles and the activation of distinct signalling cascades. For example, the level of insulin-like growth factor-binding protein 2 (*IGFBP2*), a key regulator of adipogenesis, is lower in WJ-MSCs than in AT-MSCs under standard conditions. Consequently, *IGFBP2*, by modulating JNK and Akt pathways, facilitates the faster adipose-directed transformation of AT-MSCs^1^.

In addition to gene expression and signalling regulation, the availability and composition of fatty acids within the cells can influence triglyceride synthesis and subsequent lipid droplet (LD) accumulation, thereby modulating adipogenic differentiation. It is known that pre-existing lipid reserves within human pre-adipocytes serve as substrates for LD formation during differentiation, while the fatty acid composition of these reserves directly impacts differentiation efficiency^9^. Mono- or polyunsaturated fatty acids can enhance adipogenesis via the upregulation of adipogenic genes or contribute to triglyceride biosynthesis and LD stability^10,11^. Conversely, an imbalance in saturated fatty acids may induce lipotoxic stress, impairing differentiation outcomes^12–14^. However, whether similar mechanisms exist in WJ-MSCs remains unknown, highlighting the need for dedicated studies exploring their intrinsic lipid metabolism in the context of adipogenesis.

Finally, differentiation media composition can affect the efficiency of cell transformation in the adipogenic direction. These cocktails typically include inducers such as 3-isobutyl-1-methylxanthine (IBMX), indomethacin, insulin, and dexamethasone, which act through distinct molecular mechanisms. IBMX elevates intracellular cAMP levels, activating protein kinase A signalling pathways critical for early adipogenic gene expression. Indomethacin functions as a ligand for peroxisome proliferator-activated receptor gamma (PPAR-γ), a master regulator of adipogenesis. Insulin facilitates glucose uptake and lipogenesis, while dexamethasone modulates glucocorticoid receptor-mediated transcriptional activity^10,13^.

Optimization of the adipogenic differentiation protocols for WJ-MSCs presents a promising opportunity to match or even surpass the adipogenic efficiency observed in AT-MSCs. Ozhava D. et al. demonstrated that targeted modifications of differentiation protocols could significantly improve adipogenic outcomes^15^. According to their findings, among several tested inducers, efficient differentiation was achieved by a cocktail composed of 7 fatty acids.

The current study explored the adipogenic differentiation potential of MSCs derived from Wharton’s jelly and adipose tissue. The initial lipidomic profiles of these cells were compared, focusing on triglyceride content and fatty acid composition. Additionally, we attempted to reveal the role of exogenous fatty acids in WJ- and AT-MSC adipogenic differentiation and improve the differentiation efficiency needed for broad translation in adipose tissue engineering.

## Results

### Adipogenic differentiation of WJ- and AT-MSCs in standard conditions

**Fig. 1:**
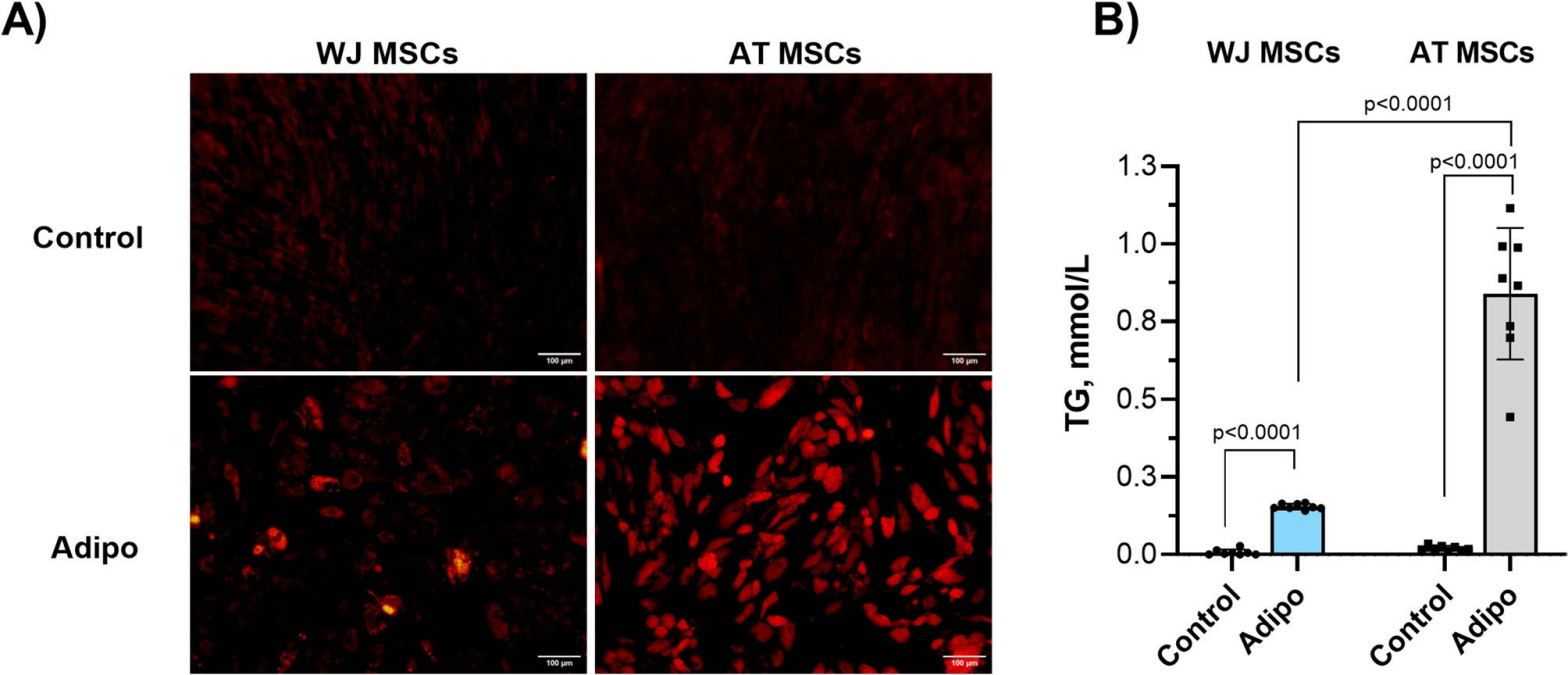
Adipogenic differentiation of WJ-MSCs and AT-MSCs (*N* = 4 for each) following the standard induction protocol: (**A**) Lipid droplets in control and induced cultures stained by Nile Red (21 days of culture); (**B**) TG accumulation in control groups and after directed adipogenic differentiation (21 days of culture). Data is presented as Mean ± SD and shown in duplicates.

At the first stage of our study, we assessed the efficacy of the adipogenic differentiation of WJ- and AT-MSCs using a common induction medium containing IBMX, dexamethasone, insulin, and indomethacin (Fig. 1).

Following 21 days of induction, a significantly lower amount of visible intracellular LDs, stained by Nile red, was observed in WJ-derived cells compared to AT-MSCs (Fig. 1A). Although signs of differentiation were detected in WJ-MSC cultures, the amount of Nile red positive cells was lower than in AT. Of note, no spontaneous differentiation was detected in both cell cultures, confirming the effect of induction.

The following quantitative analysis of the intracellular TG content showed the higher differentiation efficacy of AT-MSCs compared to WJ-derived counterparts. In WJ-MSCs, the adipogenic induction resulted in a significant increase in the TG content. However, the average level of accumulated TGs was around 3-6-fold lower compared to the induced AT-derived cells (Fig. 1B).

### Lipidomic profile of WJ- and AT-MSCs

**Fig. 2:**
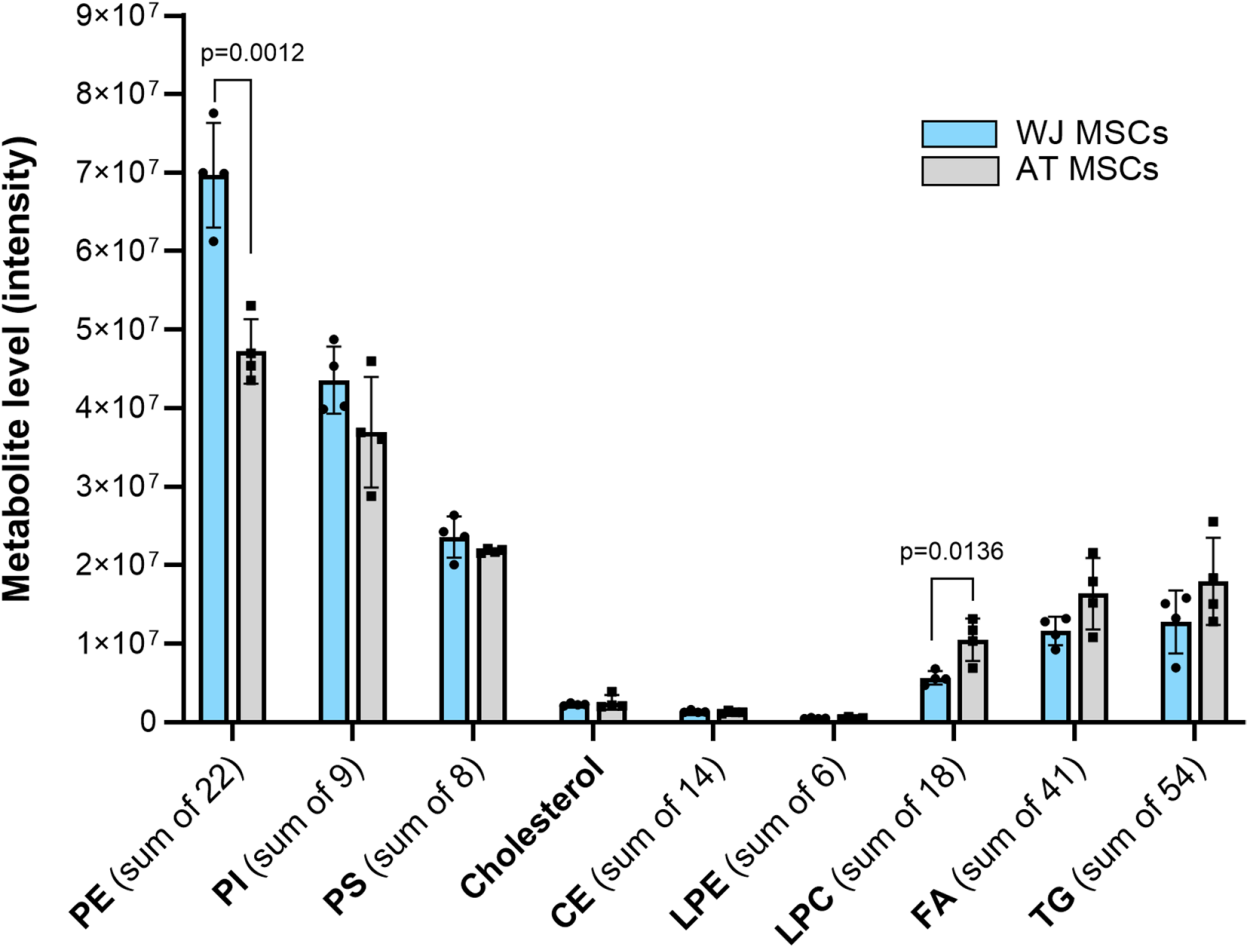
Lipidomic profiles of WJ-MSCs and AT-MSCs (*N* = 4) based on liquid chromatography-mass spectrometry (LC-MS). Abbreviations: phosphatidylethanolamines (PE), phosphatidylinositols (PI), phosphatidylserines (PS), cholesterol esters (CE), lysophosphatidylcholines (LPC), lysophosphatidylethanolamines (LPE), free fatty acids (FAs), and triglycerides (TG). Data is presented as Mean ± SD. Signal intensities were normalized to the respective total ion count (TIC) before subsequent statistical analysis.

To uncover the possible cause of these differences, we explored the lipidomic profiles of the initial non-induced cell cultures, presuming that the natural intracellular lipid composition might differ between AT- and WJ-MSCs (Fig. 2).

The overall lipid profile of WJ- and AT-MSCs was quite similar. However, a trend was observed toward a higher representation of structural phospholipids in WJ-MSCs and an enrichment of neutral lipids (TGs and FAs) in AT-MSCs. A statistically significant difference was detected only in the content of phosphatidylethanolamines (PE), which was higher in WJ-MSCs, and lysophosphatidylcholines (LPCs), which were 1.86-fold more abundant in AT-MSCs (Fig. 2). Increased LPC is an indication of elevated lipolytic activity, consistent with pre-adipogenic priming of AT-MSCs. Reduced phospholipid levels indicate that AT-MSCs may shift energy and lipid resources from membrane synthesis to TG production and storage.

**Fig. 3:**
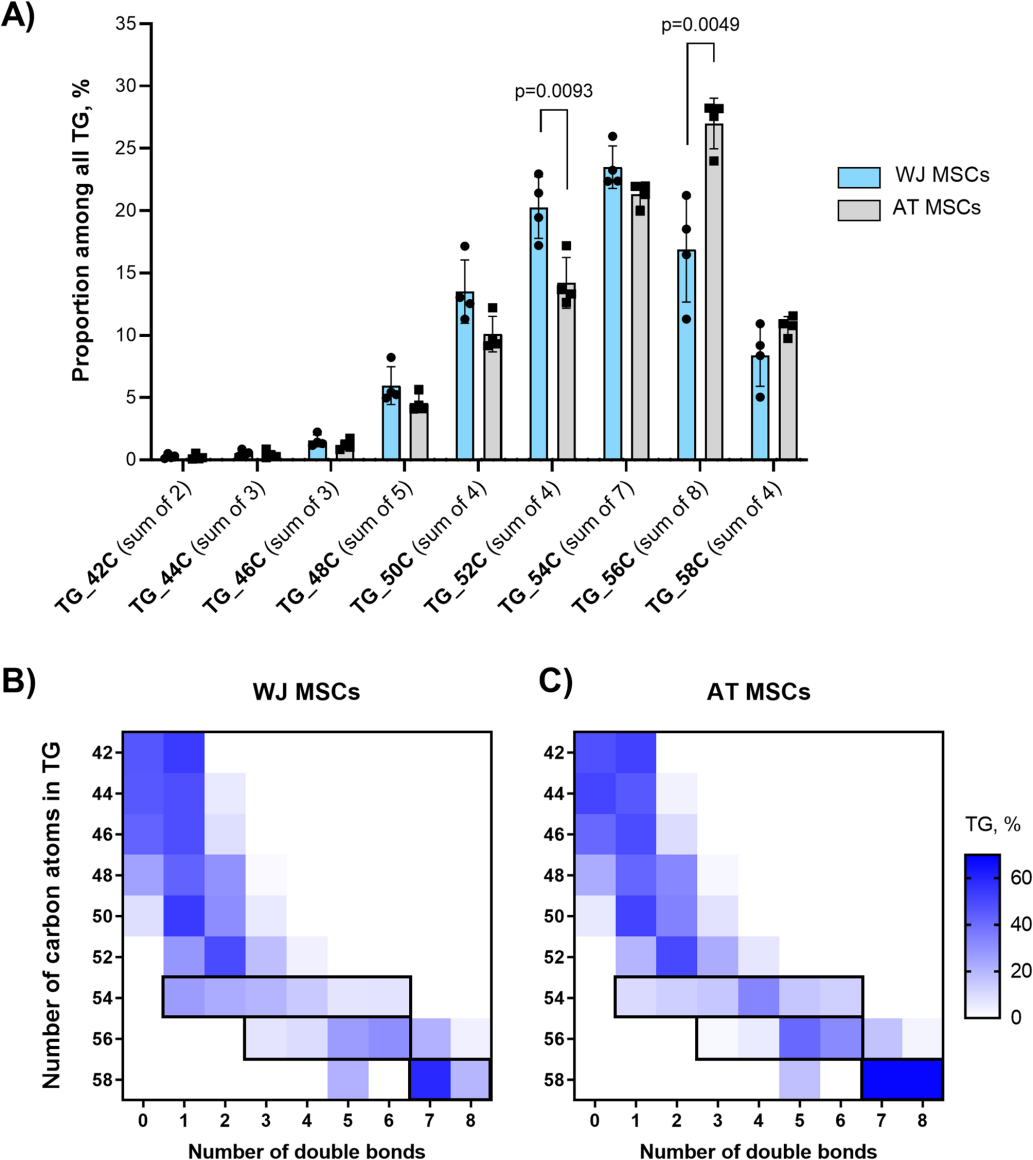
Comparison of TG proportion in WJ-MSCs and AT-MSCs (*N* = 4): (**A**) Distribution of different TG species in WJ-MSCs and AT-MSCs; (**B**,**C**) Distribution of TGs by carbon number and number of double bonds in WJ-MSCs (**B**) and AT-MSCs (**C**) (*N* = 4). The heatmap plot represents the proportion of different TG species among all TGs. The black frame highlights differences between cell types, particularly in long-chain TGs (54–58 carbons), where AT-MSCs shift towards a higher number of double bonds relative to WJ-MSCs.

**Fig. 4:**
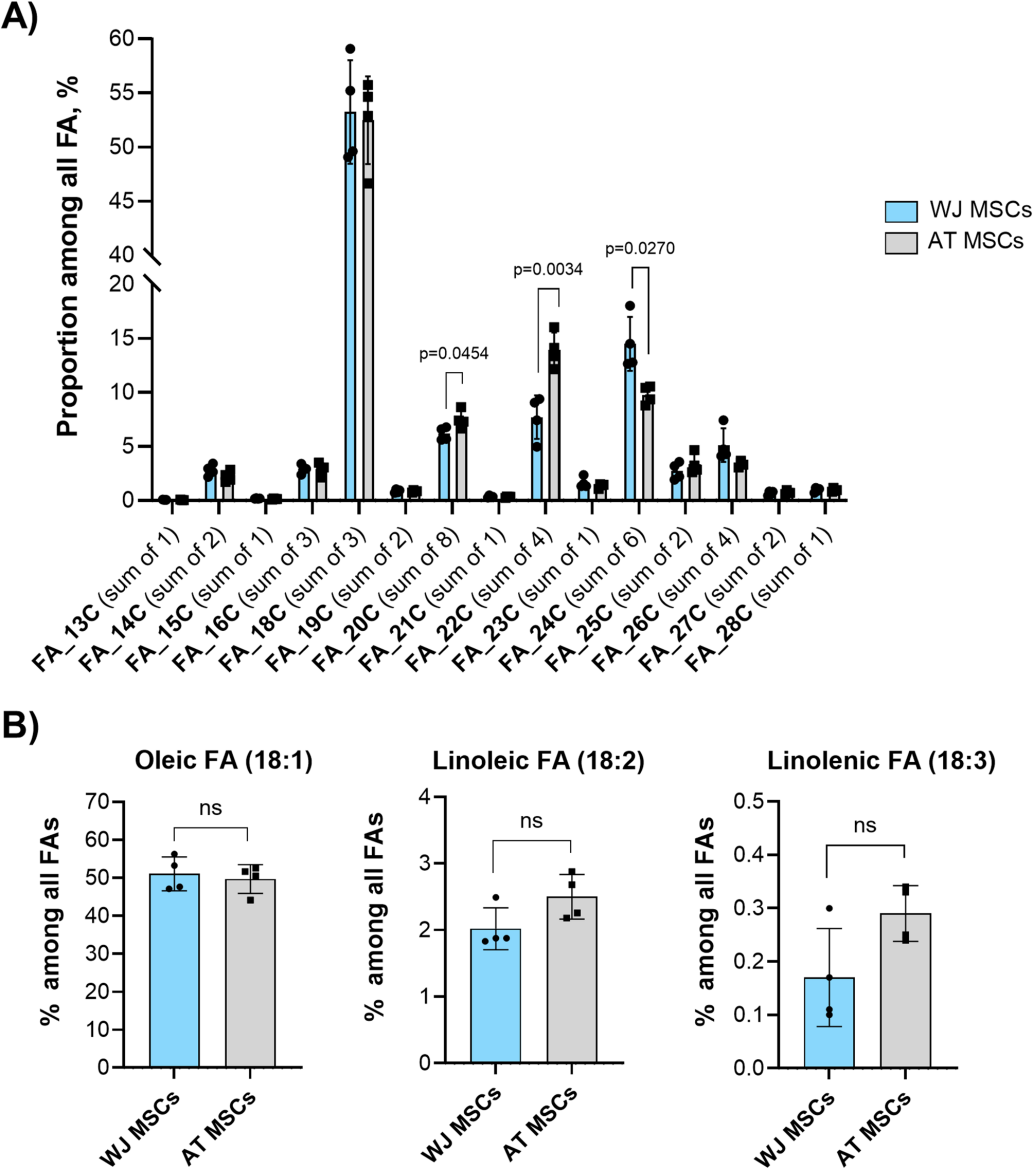
Comparison of FA proportion in WJ-MSCs and AT-MSCs (*N* = 4): (**A**) Differences in FA profile in WJ-MSCs and AT-MSCs; (**B**) Share of 18-carbon fatty acids, namely oleic acid, linoleic and linolenic acids, among all FA; Data is presented as Mean ± SD.

The following analysis of the distribution of specific TG and FA types within their families allowed us to detect differences between the WJ- and AT-MSCs (Figs. 3 and 4). The TG composition was distinct between the two cell types. In WJ-MSCs, we observed a progressive increase in the abundance of TG species with the number of carbons from 48 to 52, reaching a peak at 54 carbons, followed by a decline in content at 56 carbons and higher. In contrast, in AT-MSCs, the peak TG abundance was observed at 56 carbons, with a 10% higher relative content of these TG species than those in WJ-MSCs. However, a considerably higher relative content of TG species with 52 carbons was detected in WJ-MSCs (20.3%±2.1% vs. 14.2%±1.8%, Fig. 3A).

Next, we examined the abundance of double bonds in TGs from two cell types (Fig. 3B,C). TG species with 42–48 carbon atoms consisted predominantly of saturated fatty acids, comprising up to 40% of these molecules. In contrast, TGs with 52 or more carbon atoms contained at least one unsaturated fatty acid. The distribution of double bonds in TG species was comparable between WJ-MSCs and AT-MSCs for TGs up to 52 carbon atoms. However, long-chain TG species (54, 56, and 58 carbons) exhibited distinct differences between the two cell types. In AT-MSCs (Fig. 3C), a marked shift towards an increased number of double bonds was observed relative to WJ-MSCs (Fig. 3B), highlighting cell type-specific differences in TG saturation profiles.

Comparing the FA profiles of WJ- and AT-MSCs, the most dominant relative levels were detected for FAs containing 18 carbons (sum of 3), reaching more than 50% of total FA level in both cell types (Fig. 4). The significantly higher amount of long-chain FA20C and FA22C was detected in AT-MSCs. In contrast, the relative content of 24 C FAs was by 5% higher in WJ-MSCs (Fig. 4).

Considering the crucial role of 18 C FAs in adipogenesis and TG formation, we further assessed the relative content of FA18:1 (oleic acid, OA), FA18:2 (linoleic acid, LA), and FA18:3 (linolenic acid). No significant differences in the distribution of these FAs between the two cell types were detected (Fig. 4B). OA was the most abundant, LA accounted for only 2–3% of the total FA composition, and linolenic acid represented less than 1%. Despite the similar availability of FAs essential for adipogenesis, we hypothesized that their utilization and subsequent TG synthesis efficiency may differ between WJ-MSCs and AT-MSCs. Thus, oversaturating cells with OA and LA could serve as a strategy to enhance the esterification of glycerol, thereby promoting TG formation in WJ-MSCs.

### Effect of OA and LA supplementation on induced adipogenic differentiation of WJ- and AT-MSCs

**Fig. 5:**
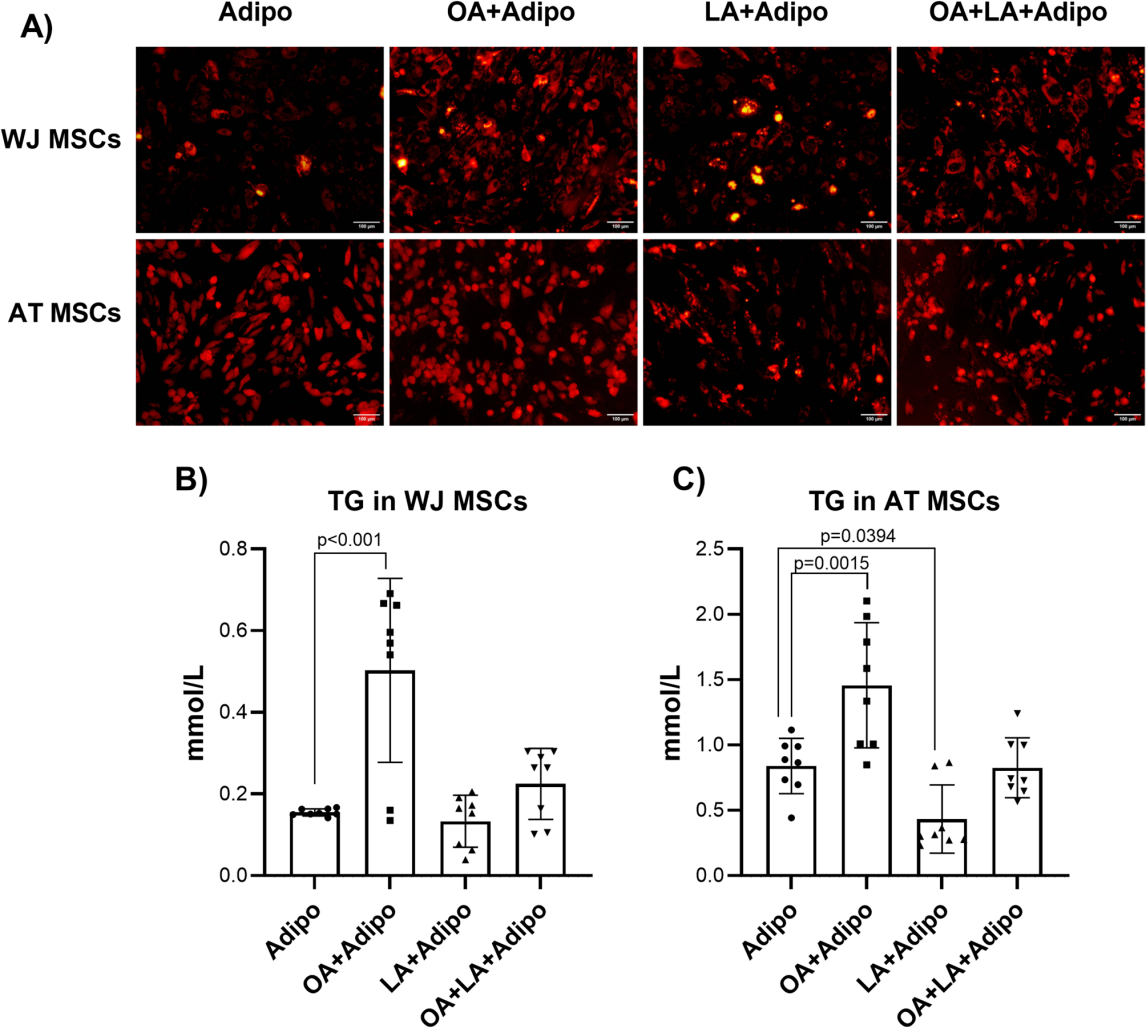
Adipogenic differentiation of WJ-MSCs and AT-MSCs (*N* = 4) following OA (100 µM), LA (100 µM), or their mixture (50 µM each) supplementation. (**A**) Lipid droplets after 21 days of induction by standard differentiation media and with FA addition, Nile red-stained cultures. (**B**) TG accumulation in WJ-MSCs following FA supplementation; (**C**) TG accumulation in AT-MSCs following FA supplementation.

To confirm the roles of OA and LA in promoting adipogenic differentiation and to improve the differentiation outcomes of WJ-MSCs, we supplemented the induction medium with exogenous FAs and cultured the cells for 21 days. Either OA or LA at a concentration of 100 µM, or their combination at 50 µM each, were applied as an additive to culture medium (Fig. 5). After 3 weeks of induction, there were significant differences in adipogenic differentiation of WJ-MSCs cultured in the presence of OA compared to FA-free conditions (Fig. 5A). The amount of Nile-red positive cells in these groups, as well as the size of intracellular LDs, was higher than after FA-free induction. Interestingly, LA supplementation did not cause any visible impact on the efficacy of adipogenic differentiation of WJ-MSCs.

In contrast to WJ-MSCs, the effect of FAs on the adipogenic differentiation of the AT-MSCs was different. The supplementation of the induction medium by OA did not have a visible positive impact on the accumulation of intracellular LDs. Moreover, adding LA suppressed the differentiation efficacy, and combined OA/LA application led to mid-range results between OA and LA groups (Fig. 5A).

The quantitative analysis of the TG content in differentiated MSCs confirmed the visual interpretations. A notable, around 4-fold increase in TG levels was observed in WJ-MSCs after 21 days of differentiation in the presence of OA (Fig. 5B). No significant effect was observed in LA-supplemented cultures or the OA/LA group.

Following the characterization of WJ MSCs, we revealed that the addition of OA to AT-MSC cultures led to a 1.7-fold increase in TG accumulation (Fig. 5C). The use of the OA/LA combination did not cause any differences in the TG content of AT-MSCs. In contrast, the culture of cells in the presence of LA alone even had a suppressive effect.

**Fig. 6:**
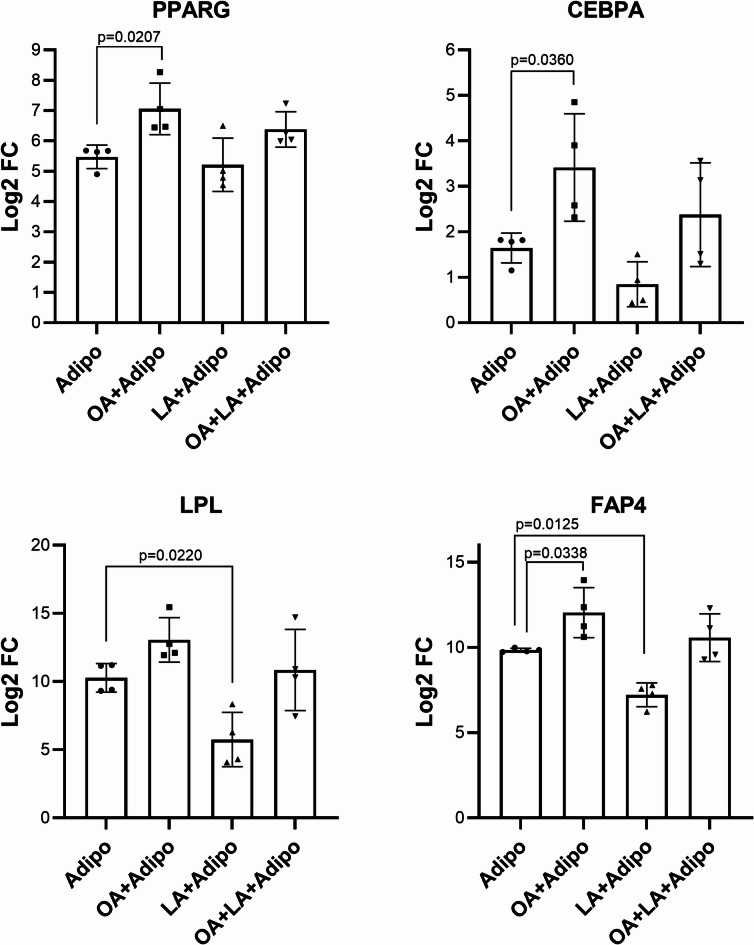
Expression of key adipogenic markers in WJ-MSCs after differentiation with and without exogenous FA supplementation (*N* = 4). Data is presented as Mean ± SD of Log2-fold change values vs. non-differentiated control cultures assessed by RT-PCR.

The effect of the exogenous FA supplementation on the gene expression of the differentiated WJ- and AT-MSCs was further analysed by qPCR using specific adipogenic markers *PPARG*,* LPL*,* CEBPA*, and *FABP4*. A notable upregulation of *PPARG*, *CEBPA*, and *FABP4* by 3-fold, 3.4-fold, and 4.6-fold, respectively, with a trend of *LPL* increase was detected after culturing WJ-MSCs in an induction medium supplemented with OA (Fig. 6). Mildly elevated levels in all studied adipogenic genes were observed in the OA/LA cultured group; however, the expression changes did not reach statistical significance (Fig. 6). Although the supplementation of the induction medium by LA alone did not result in any significant effect on the expression of *PPARG* and *CEBPA*, it led to downregulation of late adipogenic markers, namely *LPL* and *FABP4* by 23-fold and 6.1-fold.

**Fig. 7:**
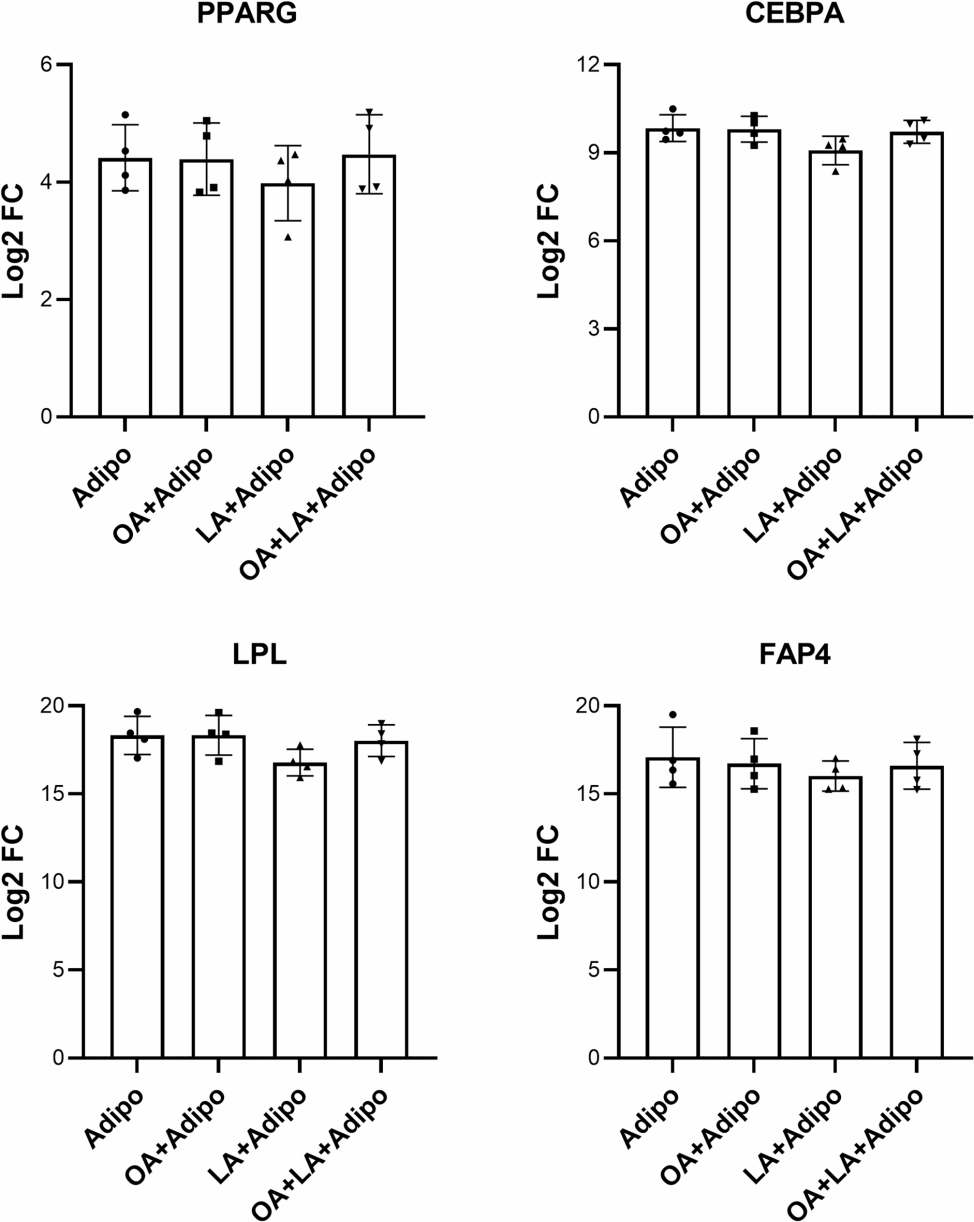
Expression of key adipogenic markers in AT-MSCs after differentiation with and without exogenous FA supplementation (*N* = 4). Data is presented as Mean ± SD of Log2-fold change values vs. non-differentiated control cultures assessed by RT-PCR.

The gene response of AT-MSCs to FA addition differed compared to WJ-MSCs (Fig. 7). The induction of adipogenic differentiation in the presence of OA did not lead to any significant changes in *PPARG*, *CEBPA* or *LPL* genes (Fig. 7) compared to FA-free conditions. Similar gene expression was detected in MSCs cultured in the presence of an OA/LA combination and LA alone.

Therefore, the effects of exogenous fatty acids on MSC adipogenesis varied depending on the specific type of fatty acid and the tissue origin of the MSCs. The OA positively affected the efficacy of the adipogenic differentiation of MSCs, independently of the source, while LA either suppressed or did not affect the MSC adipogenesis.

## Discussion

Improving the adipogenic differentiation capacity of WJ-MSCs is crucial for their potential use in regenerative medicine, particularly in soft tissue reconstruction, metabolic disease modelling, and adipose tissue engineering. WJ-MSCs offer notable translational advantages over adult tissue-derived MSCs. Harvested non-invasively from umbilical cords, an otherwise discarded perinatal tissue, WJ-MSCs are obtained through a procedure that is ethically uncontroversial and poses no risk to the donor^16^. This contrasts with the majority of other MSC sources, which require invasive harvesting procedures that may be impractical in elderly or chronically ill individuals. Moreover, WJ-MSCs are characterized by low expression of MHC class I and negligible levels of MHC class II and co-stimulatory molecules, making them less immunogenic and well-suited for allogeneic applications^17^. The use of umbilical cords from young, healthy donors also ensures a consistent and high-quality cellular product, minimizing donor-related variability commonly observed in adult-derived MSCs, where age, body mass index, and comorbidities can significantly influence cell phenotype and function^18,19^.

Understanding the differences between WJ-MSCs and cells committed to adipogenesis, such as AT-MSCs, is essential for developing effective strategies to enhance adipogenic potential. In this study, we compared the lipidomic profiles of WJ-MSCs and AT-MSCs. We demonstrated for the first time that the initial TG composition of WJ-MSCs and AT-MSCs differs significantly, potentially contributing to their distinct adipogenic capacities. Additionally, we showed that WJ-MSC adipogenesis can be improved by supplementing the culture medium with exogenous OA.

In the current study, we have observed a significantly lower accumulation of TGs in response to standard adipogenic induction in WJ-MSCs. The distinct differentiation efficacy between AT- and WJ-MSCs has been previously shown in other reports^20–22^. For example, reduced adipogenesis of porcine WJ-MSCs was detected in comparison with AT-derived cells^20^ or with BM-MSCs of rabbits^21^ and human^22^. Similarly, lower adipogenic differentiation capacity of WJ-MSCs was reported when compared to periodontal ligament stem cells^1^. We presumed that the initial lipidomic profile could be responsible for these differences and performed the lipidomic analysis of the cell cultures, focusing on the TG and FA content in non-induced cells.

Indeed, TG composition was distinct between these two cell types. We detected a higher relative content of TG with 52 carbons in WJ-MSCs, while TG56C species prevailed in AT-MSCs. The size of TG56C is greater than that of TG52C, which may result in a faster accumulation of the critical mass and subsequent initiation of lipid lens formation, followed by coalescence, growth, and budding from the endoplasmic reticulum^23^. In contrast, smaller TGs likely require more time to reach the critical mass necessary for LD nucleation, potentially contributing to a slower rate of adipogenic differentiation.

Our findings confirm that in the case of AT-MSCs, LD growth was already visible after 7 days of induction, followed by further enhancement over time. However, we did not notice sufficient adipogenic differentiation in WJ-MSCs even after 21 days of culture using a standard induction cocktail, which may suggest a delayed or impaired capacity for LD formation in these cells. Such observations were in line with our hypothesis that the pre-existing intracellular lipid reserves may determine the further adipogenic differentiation efficacy of the cells.

Although there are several publications showing the role of lipidomic landscape in regulating the functional properties of MSCs, including immunomodulation and differentiation^24–26^, data on how lipidomic profiles vary between MSCs of different tissue origins remains limited. In a recent study, the sphingolipid composition of MSCs derived from bone marrow, adipose tissue, and umbilical cord tissue was compared^24^. The authors detected higher levels of sphingomyelins in adipose tissue MSCs, while the glycosphingolipids (HexCer and LCB So1P) were more abundant in umbilical cord tissue-derived cells. These findings support the concept of lipidomic tissue specificity, suggesting that MSCs may partially retain the lipid characteristics of their tissue of origin. In another study, Burk et al. compared the phospholipid profiles of human and equine AT-MSCs with human fibroblasts and peripheral blood mononuclear cells (PBMC)^27^. MSCs exhibited greater diversity in the phospholipid species compared to PBMC and fibroblasts. Notably, several lipids, such as phosphatidylethanolamine (PE O-36:3) and phosphatidylglycerol (PG 40:7), were identified as potentially MSC-specific markers, as they were absent in fibroblasts. Nevertheless, to our knowledge, a direct comparison of TG and FA profiles between the AT- and WJ-MSCs has not been previously reported.

The availability of specific free fatty acids as building blocks for TG synthesis determines the final TG structure. OA, LA, and palmitic acid (PA) are the most abundant fatty acids esterified to TGs in human cells^14,28–31^. The significant content of free saturated PA has been shown in differentiated AT-MSCs^14,29^ and differentiated and non-induced BM-MSCs^30,31^. In our study, the relative share of FA16C within the total FA level was negligible in both WJ- and AT-MSCs and did not reach 5%. Interestingly, we also did not observe significant variation in the contents of free FA18C molecules in WJ- and AT-MSC cultures (Fig. 3C). We presume that longer (≥ 18 C) chain fatty acids may contribute to the synthesis of larger TG species (such as TG56C), which could partly explain the formation of larger LDs in AT-MSCs compared to WJ-MSCs. Previous studies have shown that arachidonic acid (FA22:4) supported the formation of larger LDs in AT stromal vascular fraction cells^32^. Additionally, elevated levels of FA22C, initially and after adipogenic differentiation, have been reported in rat BM-MSCs^30,31^. In our study, we also detected a higher share of FA22C in AT-MSCs than in WJ-MSCs.

The differences in the size of TG molecules revealed in our study for WJ- and AT-derived cells and their distinct efficiency of LD formation most likely reflect different energy and metabolic priorities. WJ-MSCs are highly plastic cells with fast proliferation rates, while AT-MSCs have intrinsic adipogenic priming and are more specialized for storing energy in the form of TGs^3,5,6^. Therefore, we tried to shift WJ-MSC’s metabolic profile by enriching the media with additional exogenous substrates for TG synthesis, such as OA and LA, to improve their adipogenesis efficacy. Indeed, adding OA to the culture medium significantly enhanced LD formation in WJ-MSCs. The total TG content nearly reached the levels observed in AT-MSCs differentiated without exogenous FAs. Besides the increased LD formation, adding OA drove the upregulation of adipogenic genes *PPARG*, *CEBPA*, and *FABP4* in WJ-MSCs. Comparable results for the same set of genes were shown for porcine stromal-vascular cells^33^. The upregulation of *PPARG* in response to OA supplementation was reported in other studies^34^.

In AT-MSCs, total TG content was also increased in the OA-supplemented group. Yet, no significant impact of the OA addition on the gene expression profile was detected. The evidence suggests that in the case of AT-MSCs, the exogenous OA acts only as a building block for the TG formation. In contrast, in the case of WJ-MSCs, it has a dual effect, additionally driving the adipogenesis at the gene level via PPARG pathway upregulation. It confirms the different responses of these two cell types to adipogenic inducers. The positive impact of exogenous OA on the adipogenic differentiation of MSCs from other tissues or preadipocytes has already been confirmed in several studies^12,30,31^. A substantial increase in LD formation was also observed for FA mixtures containing OA^13,15^.

Contrary to expectations, adding LA to the culture medium in our study suppressed LD formation compared to FA-free cultures. The effects of LA on induced adipogenesis remain controversial. Turner et al. reported a significant increase in TG content after post-induction maturation of AT-MSCs in the presence of OA and LA under both 2D and 3D conditions^34^. However, the other studies demonstrated that culturing the 3T3-L1 cells in the presence of LA or arachidonic acid inhibited TG accumulation^35,36^. Similar findings were reported for porcine AT stromal vascular fraction cells^33^, where incubation for 5 days with LA resulted in cell detachment.

The differential effects of LA and OA on adipogenesis can be attributed to their distinct biochemical properties and roles in cellular metabolism. OA is a monounsaturated FA and can be readily incorporated into TGs. LA is a polyunsaturated FA and, therefore, less stable and may undergo lipid peroxidation, leading to oxidative stress^37^. Moreover, in both cell types studied in this paper, the share of OA among all FA reached around 50%, while only around 2-2.5% belonged to LA. Interestingly, several studies^14,30,31^ have reported a notable difference in LA content between native adipose tissue and differentiated MSCs. While LA constitutes a significant portion of the fatty acid profile in adipose tissue, its levels remain low in MSCs both before and after adipogenic differentiation.

The next step will be to translate these findings into a 3D environment to mimic physiological conditions better and assess their functionality in clinically relevant models. Further studies are needed to evaluate the lipidomic profile of differentiated WJ-MSCs and their correspondence to native adipose tissue, along with the long-term stability of the induced adipogenic phenotype and its potential for integration into regenerative therapies.

## Conclusions

We demonstrated the differences between the efficiency of adipogenic differentiation of WJ- and AT-MSCs, most likely associated with the initially distinct TG profile and the response level to the standard inducers. The adipogenic differentiation of WJ-MSCs can be improved by supplementing the culture medium with monounsaturated OA. The optimized method for adipogenic differentiation of WJ-MSCs can be applied to improve the therapeutic performance of these cells for soft tissue engineering.

## Methods

### Isolation of WJ-MSCs

Discarded human umbilical cords were obtained at the University Hospital Plzen (Plzen, Czech Republic) from healthy full-term neonates (*N* = 4) after spontaneous delivery, according to all ethical guidelines. About 10 cm of the umbilical cord was aseptically collected, placed in sterile PBS with antibiotic–antimycotic solution at 4 °C, and transported to the laboratory within 24 h. After washing several times in PBS and brief exposure to 10% Betadine (EGIS Pharmaceuticals PLC, Budapest, Hungary), blood vessels were removed, the remaining Wharton’s jelly tissue was chopped into small fragments and the cells were isolated by enzymatic digestion in PBS-AA solution containing 0.26 U/ml Liberase-TM™ (Roche Custom Biotech, Mannheim, Germany) and 1 mg/ml hyaluronidase at 37 °C with constant shaking for 2 h. After removing undigested fragments with 40-µm cell strainers, cells were centrifuged at 450 × g for 10 min^6,38^.

### Isolation of AT-MSCs

AT samples were obtained from healthy volunteers (*N* = 4) who had undergone liposuction procedures for aesthetic reasons and processed as previously described^6^. Briefly, the lipoaspirate was repeatedly washed in PBS and enzymatically digested by 0.3 PzU/ml collagenase type I (Thermo Fisher Scientific) at 37 °C for 2 h with constant shaking. After centrifugation at 200 × g for 10 min, the stromal vascular fraction was washed twice with PBS and expanded.

### Cell culture and expansion

Primary cell suspensions were diluted in a complete culture medium containing α-MEM (Gibco^®^, Thermo Fisher Scientific), 5% human platelet lysate (HPL, Bioinova a.s.), penicillin/streptomycin (Gibco^®^, Thermo Fisher Scientific), and GlutaMAX (Gibco^®^, Thermo Fisher Scientific). Cells were cultured at 37 °C in a humidified atmosphere with 5% CO_2_. Regular media changes were done twice a week. After reaching near-confluence, cells were harvested using the 0.05% Trypsin/EDTA solution (Gibco^®^, Thermo Fisher Scientific) and reseeded onto a fresh plastic surface (Nunc, Roskilde, Denmark) at a density of 5 × 10^3^ cells/cm^2^. Cells in passage 2 were cryopreserved in culture medium, supplemented with 10% DMSO, using a 1 °C/min cooling rate down to 80 °C, and stored at -196 °C until further use. Before use, the cryopreserved cell cultures were thawed at 37–40 °C and additionally expanded in the α-MEM medium containing GlutaMAX, supplemented with 10% fetal bovine serum (FBS), 100 units/mL of penicillin, and 100 µg/mL of streptomycin (all from Gibco^®^, Thermo Fisher Scientific). Cells from passages 3–5 were used for subsequent experiments.

### Adipogenic differentiation

For adipogenic differentiation, AT- and WJ-MSCs were cultured for 21 days in a medium composed of α-MEM, containing 10% FBS, penicillin/streptomycin, GlutaMAX, 0.5 mM 3-isobutyl-1-methylxanthine, 0.1 µM dexamethasone, 0.1 mM indomethacin, and 10 µg/ml insulin (all from Merck KGaA, Darmstadt, Germany). The medium was replaced every 3 days.

In separate experiments, the induction medium was additionally supplemented by 100 µM Oleic Acid (OA, Merck KGaA), 100 µM Linoleic acid (LA, Merck KGaA), or their mixture (50 µM each). Before the application, OA and LA were dissolved in DMSO and then complexed with albumin in FBS, resulting in an estimated BSA: FA ratio of ~ 2.4:1 to minimize toxicity while ensuring efficient binding and a minimal amount of unbound fatty acids. The final DMSO concentration during cell culture comprised 0.1%. After 21 days of culture, WJ-MSCs were harvested by trypsinization and centrifuged. The pellets were washed once with PBS and stored at − 80 °C for subsequent qPCR analysis. The other part of cell cultures was used for Nile red staining and TG content determination.

### Nile red staining and fluorescence microscopy

MSCs were fixed in a 4% buffered formalin for 30 min at 4 °C and stained with a Nile Red (1 µg/ml in PBS) solution (Merck KGaA) according to the manufacturer’s instructions. The cells were assessed using a fluorescent microscope (Leica, Germany).

### Determination of intracellular triglyceride content

The triglyceride (TG) content in AT- and WJ-MSC culture after adipogenic differentiation was determined using the Triglycerides Quantification Kit (Thermo Fisher Scientific) according to the manufacturer’s instructions. Briefly, the adherent cells were washed twice with PBS and lysed using the lysis buffer provided in the kit, followed by incubation on ice for 10 min to ensure efficient cell disruption. The lysates were centrifuged at 12,000 × g for 10 min at 4 °C, and the supernatants were collected for analysis. Triglyceride levels were measured using a colorimetric assay in the Tecan Infinite 200 (Tecan, Männedorf, Switzerland) microplate reader at 540 nm absorbance. TG concentrations were calculated from a standard formula:

$$\:TG\:\left(\frac{mmol}{L}\right)=\:\frac{ODSample\:-\:ODBlank}{ODStandard\:-\:ODBlank}\times\:Cstand\:\times\:f$$

where

Cstand: Concentration of standard (2.26 mmol/L);

f: Dilution factor of the sample before the test (equals 1 in our experiments).

The data was presented as Mean ± SD of 4 independent donor samples assessed in duplicates.

### Analysis of lipidomic profile

The lipidomic profile of cells was explored by the liquid chromatography-mass spectrometry (LC-MS) in the Service Department of Metabolomics of the Institute of Physiology of the CAS, Prague, Czech Republic. Briefly, cells were grown on 6-well plates, treated as required, quickly washed with PBS, snap-frozen, and stored at − 80 °C. Metabolites were extracted using a biphasic solvent system of cold methanol, methyl tert-butyl ether, and water^39^.

An aliquot of the bottom (polar) phase was collected and cleaned using an acetonitrile/isopropanol mixture. After evaporation, the dry extract was resuspended in 5% methanol with 0.2% formic acid, followed by separation in an Acquity UPLC HSS T3 column (Waters, Milford, MA, USA). Another aliquot of the bottom phase was evaporated, resuspended in an acetonitrile/water mixture, and separated in an Acquity UPLC BEH Amide column. Metabolites were detected in negative and positive electrospray ion mode (Thermo Q Exactive Plus instrumentation)^40^. Signal intensities were normalized to the respective total ion count (TIC) before subsequent statistical analysis.

### Quantitative real-time PCR

To assess the efficacy of adipogenic differentiation, specific human marker genes were selected:

- *CEBPA*(CCAAT/enhancer-binding protein alpha, Hs.PT.58.4022335.g),
- *PPARG*(Peroxisome proliferator-activated receptor gamma, Hs.PT.58.25464465),
- *FABP4*(Fatty Acid Binding Protein 4, Hs.PT.58.20106818),
- *LPL*(Lipoprotein lipase, Hs.PT.58.45792913).

Total RNA was extracted from cell pellets using the Total RNA Purification Kit (Norgen Biotek Cor., Canada), following the manufacturer’s protocol. The concentration and purity of RNA were assessed using a NanoDrop™ 2000/2000c Spectrophotometer (Thermo Fisher Scientific). The RNase-Free DNase I Kit (Norgen Biotek Corp., Canada) was used to improve RNA purity. RNA samples with an absorbance ratio (A260/A280) between 1.9 and 2.1 were used for subsequent experiments.

For the quantitative conversion of RNA into single-stranded cDNA, we applied a High-Capacity cDNA Reverse Transcription Kit (Thermo Fisher Scientific). RNA samples were normalized to the same amount of input RNA for reverse transcription. Reverse transcription was performed using the C1000 Touch™ Thermal Cycler (Bio-Rad, USA) set to these conditions: 25 °C for 10 min, 37 °C for 120 min, 85 °C for 5 min, and 4 °C for the end.

Quantitative real-time PCR was carried out using the CFX384 Touch Real-Time PCR Detection System (Bio-Rad, USA) following these cycling conditions: initial denaturation at 95 °C for 30 s, followed by 44 cycles of denaturation at 95 °C for 5 s, and annealing at 60 °C for 30 s extension for 72 °C for 10 s.

The following primer pairs (PrimeTime^®^ qPCR Primers, IDT) were used in the study:

**Table:** 

Genes	Forward primers (5´-3´)	Reverse primers (5´-3´)
*CEBPA*	CCACGCCTGTCCTTAGAAAG	CCCTCCACCTTCATGTAGAAC
*PPARG*	GTTTCAGAAATGCCTTGCAGT	GGATTCAGCTGGTCGATATCAC
*LPL*	GAGAAGCTATCCGCGTGA	CCTTGGAACTGCACCTGTAG
*FABP4*	ACTTGTCTCCAGTGAAAACTTTG	ATCACATCCCCATTCACACT
*PPIA*	GTGGCGGATTTGATCATTTGG	CAAGACTGAGATGCACAAGTG

The expression of target genes was normalized to *PPIA*, which was selected as the housekeeping gene due to its stable expression across conditions.

### Statistical analysis

Multivariate analysis of lipidomic data was performed in MetaboAnalyst 5.0^29^. Shares of fatty acids and various TG species in non-induced WJ-MSCs and AT-MSCs were compared using an unpaired t-test. For multiple group comparisons in experiments involving induced cell cultures, ordinary one-way ANOVA followed by Sidak’s post hoc test was performed. Data analysis and visualization were done using Prism 10.2.3. Statistically significant p-values are presented in figures as follows: *p < 0.05, **p < 0.01, ***p < 0.001, ****p < 0.0001.

## Data Availability

The data supporting this study’s findings are openly available on Zenodo at [https://doi.org/10.5281/zenodo.14773061](https:/doi.org/10.5281/zenodo.14773061) . The repository includes all figures and raw and processed metabolomics data used in this research.

## Abbreviations

AT: Adipose tissue
CE: Cholesterol-esters
FA: Fatty acids
FBS: Fetal bovine serum
IBMX : 3: Isobutyl-1-methylxanthine
IGFBP2: Insulin-like growth factor-binding protein 2
LA: Linoleic acid
LD: Lipid droplet
LPC: Lysophosphatidylcholines
LPE: Lsophosphatidylethanolamines
MSC: Multipotent mesenchymal stromal cell
OA: Oleic acid
PPARG: Peroxisome proliferator-activated receptor gamma
PUFAs: Polyunsaturated fatty acids
TG: Tiglycerides
WJ: Wharton’s jelly
